# Bridging glycated hemoglobin with quality of life and health state; a randomized case–control study among type 2 diabetes patients

**DOI:** 10.1186/s13098-018-0325-6

**Published:** 2018-03-27

**Authors:** Syed Wasif Gillani, Irfan Altaf Ansari, Hisham A. Zaghloul, Mohi Iqbal Mohammad Abdul, Syed Azhar Syed Sulaiman, Mirza R. Baig

**Affiliations:** 1 Department of Clinical & Hospital Pharmacy, College of Pharmacy, Taibah University, PO Box: 41411, Al-Madinah Al Munawarrah, Saudi Arabia; 2 Department of Pathology, College of Medicine, Taibah University, Al-Madinah Al Munawarrah, Kingdom of Saudi Arabia; 3 School of Pharmaceutical Sciences, Universiti Sains Malaysia (USM), Penang, Malaysia; 4 Dubai Pharmacy College, Dubai, UAE

**Keywords:** HRQOL, Type 2 diabetes mellitus, Case–control study, HbA1c

## Abstract

**Background:**

The aim of this study was to explore the predictors of QOL and health state and examine the relationship with glycemic control among type 2 diabetes mellitus (T2DM) patients.

**Methods:**

A randomized cross-sectional case–control study was conducted among n = 600 T2DM patients of Malaysia. Study population was distributed into three groups as: controls: patients with HbA1c ≤ 7 (n = 199), cases arm 1: with HbA1c 7–7.9 (n = 204) and cases arm 2 (n = 197): with HbA1c ≥ 8 consecutively last 3 times.

**Results:**

Participants with diabetes history > 10 years exhibits higher mean QOL score among all the three groups. In contrast mean health status score significantly (*p *< 0.001) reduced with the exposure duration of diabetes both within and intergroup assessment that participants with poor glycemic control (arm 2) had significantly higher mean QOL score with knowledge and self-care dimensions as compared to others, however mean health state scores were significantly (*p *< 0.001) lower in all assessment dimensions as compared to controls. The *F* test of significance showed that demographic and clinical parameters were strong predictors of QOL, whereas self-care activities, comorbidities, ability of positive management and BMI were significant predictors to health state for consistent glycemic control (controls) as compared to poor glycemic control (arm 2) participants.

**Conclusion:**

This study suggested that poor glycemic index reported low self-care behavior, increase barriers to daily living activities and poor ability to manage diabetes positively, which cause poor QOL and decrease health state.

## Background

Diabetes mellitus is the chronic progressive metabolic disorder associated with severe morbidity and mortality [1]. International Diabetes Federation predicted 642 million patients with diabetes by 2040 [2]. Type 2 diabetes (T2DM) is the most common type of diabetes all over the world. There are several long term clinical complications associated with T2DM [3] which will affect the patient’s well-being and general health state [4]. Therefore, T2DM is considered as a major threat to public health [5]. Early incidence of T2DM in adults increased the risks of both micro and macrovascular complications which in term lead to poor health-related quality of life [6–8].

Health-related quality of life (HRQOL) is a multidimensional construct consisting of physical, social, emotional, role functioning etc. [9]. Diabetes management focus on multifactorial concept, several demographic, clinical as well as behavioral factors influence the patient-specific disease outcomes [7, 10]. Glycemic control is one of the ultimate tools to evaluate the patient’s disease progression and treatment success [3, 6] however, clinical literature showed that not only treatment but patient’s perception and self-care behavior are also strong mediating factors for glycemic control among T2DM patients [3, 6, 8, 11]. Several studies have shown positive effect of self-care management to glycemic index [4, 7, 9–11], also diabetic-related knowledge is considered as an important factor for development/improvement in self-care behavior and practices [12]. Similarly, chronic or long term complications reduce the well-being of patient with adverse changes on self-care behavior thus resulted with poor HRQOL and decrease health status/state [13, 14]. But none of previous studies identified the predictors of QOL and health state among different glycemic control levels. It is important to determine the predictors of QOL and health state with consistent to poor glycemic control type 2 diabetes mellitus patients. Hence, the aim of this study was to explore the predictors of QOL and health state and examine the relationship with glycemic index among T2DM patients.

## Methods

### Ethic approval

This study has attained the ethics approval from ministry of health Malaysia and clinical research committee (CRC) of the hospital.

### Study design

A randomized cross-sectional case–control (two-case arm groups) study was conducted among type 2 diabetes mellitus (T2DM) patients of Malaysia. Participants were identified and enrolled from diabetic clinic of governmental tertiary care hospital in Kuala Lumpur, Malaysia.

### Sample and sampling procedure

**Fig. 1:**
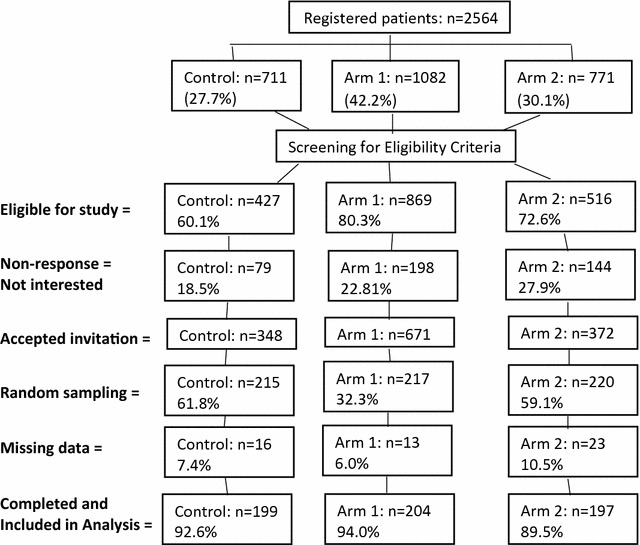
Patient recruitment process

A complete list of the registered patients with T2DM was derived from the electronic system of diabetic clinic with total sample of 2564 (Fig. 1). Selection and screening process is presented in Fig. 1. The inclusion criteria for sample population were; patients age at diagnosis should be > 30 years, under treatment for at least 2 years and able communicate and understand either in Malay or English language.

Should not have severe clinical condition, e.g., end-stage renal failure, liver cirrhosis, cancer (any type), physical disability, mental impairment/condition. And also special population; pregnant women, geriatric patients (age > 75 years), patients reporting using herbal medications for diabetes mellitus.

Sample size was estimated with G*power software at power of 0.95 and SD of 1% on 2-tailed significance testing [15]. Assuming non-response rate about 20%, a minimum of 180 participants/group were required to achieve 95% power. A random sampling method was applied to enroll patients by using random number tables in excel software.

### Controls and cases arms

Controls: patients with HbA1c ≤ 7 consecutively last 3 times (consistent glycemic index).

Cases arm 1: patients with HbA1c 7–7.9 consecutively last 3 times (moderate glycemic index).

Cases arm 2: patient with HbA1c ≥ 8 consecutively last 3 times (poor glycemic index).

### Measurement tools

Three different instruments were used to collect data from participants of this study.

*Sociodemographic and clinical baseline tool* A self-developed data collection form was used to collect participants related demographic data and relevant physiological characteristics.

*Health-related quality of life (HRQOL)* Euro-QOL (EQ-5D-5L) [16] was used to evaluated QOL, it consists of five-dimensions and each with 5-likert scale response. The EQ Visual analog scale (VAS) (10-point interval) was used to record participants self-rated health status; 0 reflects ‘worst imaginable health’ at bottom and 100 represents ‘best health’ at top. The VAS score and 5-dimension index score converted to value score between 0 and 1 (worst health-best health state) [17].

*Diabetes self-care activities scale (DSCA)* [18] used to evaluate the self-care activities among the participants. The DSCA is a self-reporting tool consists of 6-item subscale including; diet, exercise, serum glucose monitoring, foot care, medication adherence and smoking for the past 7 days. For all the item 1–18, use the number of days per week on a scale of 0–7 and mean the score in reverse pattern as; 0 = 7, 1 = 6, 2 = 5, 3 = 4, 4 = 3, 5 = 2, 6 = 1, 7 = 0.

### TOOL validation and reliability testing

The DSCA and EQ-5D tools were translated to Malay language incase participants can’t respond to English version. Forward- background translation method was used for Malay language and checked with monolingual testing. No discrepancies were found in the linguistic version of the instruments. The final tools were then administered in the pilot study consisting of 45 T2DM patients twice in a 3-week interval.

A pilot study (n = 45) among Malaysian T2DM patients was conducted to validate EQ-5D subscales as; mobility (Cronbach’s α coefficient = 0.89), self-care (α = 0.85), usual activities (α = 0.94), pain/discomfort (5-items, α = 0.91), and anxiety/depression (α = 0.83). Overall Cronbach’s α coefficient 0.88 was reported for the tool. Interterm correlation pattern had a range of *r* = 0.69–0.84 (mean = 0.77) for DSCA subscales. Cronbach’s α coefficient values for DSCA subscales were; diet (5-item, α = 0.84), exercise (2-items, α = 0.87), blood sugar monitoring (2-items, α = 0.91), foot care (5-items, α = 0.93), smoking (1-item, α = 0.95) and medication adherence (3-items, α = 0.88). Overall Cronbach’s α coefficient 0.90 was reported for DSCA.

### Data collection process

Data was collected by using validated ED-5D and DSCA tools from January to July 2017 among 600 patients with T2DM from diabetic clinic of governmental hospital. The study information sheet along with informed consent form was provided to all the enrolled participants. They were required to sign the consent form at the time of enrollment.

Study measurements and clinical assessment forms were labeled with a unique identifier. Principle investigator kept the coded data files separately from the code list to retain anonymity of the study population.

### Statistical analysis

Statistical package for social sciences (SPSS 21^®^) was used to evaluate the study data. Bivariate and multivariate models were used to determine the associated between gender and demographic factors and independent variables. Both normality and multicollinearity tests were performed. The QOL dimensions and health status were analyzed with ANNOVA and multivariate generalized linear model (GLM/MANOVA) [19]. A probability of < 0.05 was considered statistically significant.

## Results

A total of 600 participants were included in the study consisting of three group as; control (n = 199), arm 1 (n = 204) and arm 2 (n = 197). About 52 participants (16 = control, 13 = arm 1 and 23 = arm 2) with missing data were excluded from analysis.

### Sociodemographic and clinical parameters

No significant difference was found among gender. Both men and women were equally distributed among three groups. Majority of (67.3%) participants in control group were 35–44 years of age group; which is significantly (*p *< 0.01) higher as compared to arms 1 and 2. Arm 2 have significantly (*p *< 0.02) high percentage of participants above 65 years of age as compared to control and arm 1.

**Fig. 2:**
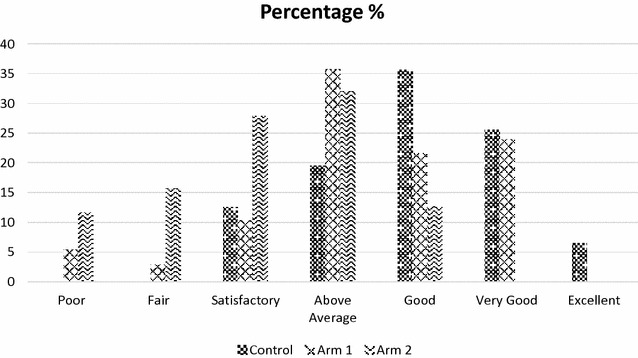
Diabetes knowledge assessment among study population (control-n = 199, arm 1 − n = 204 and arm 2 − n = 197 participants)

Marital status, education status and economics status were not significantly different with both within and inter-group assessment (Table 1). Clinical parameters showed significant association with duration of diabetes and comorbidities only. Both BMI and treatment were not significantly affecting any group. The highest frequency of adherence to self-care activities was 61.8% with 1–3 days in control group which was significantly (*p *< 0.001) higher as compared to arm 1 (44.6%) and arm 2 (7.6%). Prior diabetes education had no significant effect on glycemic control in between groups. Table 1 presented the detail findings of demographic and clinical parameters among the study population. Findings showed that participants in control group had significantly (*p *< 0.001) better diabetes knowledge as compared to participants of arm 1 and arm 2 (Fig. 2).

**Table 1:** Sociodemographic and clinical characteristics among study population

Characteristics	Control (n = 199)	Arm 1 (n = 204)		Arm 2 (n = 197)		Arms 1 vs 2
	n (%)	n (%)	*p* ^ł^	n (%)	*p* ^ł^	*p*-value
Demographics						
Gender						
Men	81 (40.7)	94 (46.1)	0.34	99 (50.2)	0.03	0.51
Women	118 (59.3)	110 (53.9)		98 (49.8)		
Age (years)						
35–44	134 (67.3)	25 (12.3)	0.01	18 (9.1)	0.00	0.02
45–54	54 (27.1)	78 (38.2)		77 (39.1)		
55–64	11 (5.6)	94 (46.1)		63 (31.9)		
65–74	–	7 (3.4)		39 (19.8)		
Marital status						
Currently married	172 (86.4)	168 (82.4)	0.48	179 (90.9)	0.71	0.63
Single/ex-married	27 (13.6)	36 (17.6)		18 (9.1)		
Education status						
Primary	13 (6.5)	17 (8.3)	0.44	11 (5.6)	0.29	0.33
Secondary	54 (27.1)	53 (26.0)		65 (33.0)		
University	103 (51.8)	97 (47.5)		80 (40.6)		
Higher	29 (14.6)	37 (18.1)		41 (20.8)		
Economic status/month						
≤ 1500 US$	67 (33.7)	70 (34.3)	0.65	76 (38.6)	0.38	0.41
> 1500 US$	132 (66.3)	134 (65.7)		121 (61.4)		
Clinical parameters						
Duration of diabetes (years)						
0–5	110 (55.3)	21 (10.3)	0.01	15 (7.6)	0.01	0.57
6–10	74 (37.2)	97 (47.5)		73 (37.1)		
> 10	15 (7.5)	86 (42.2)		109 (55.3)		
BMI (kg/m^2^)						
< 18.5/underweight	63 (31.7)	65 (31.9)	0.39	71 (36.0)	0.61	0.89
18.5–24.9/healthy	100 (50.3)	91 (44.6)		95 (48.2)		
25.0–29.9/overweight	36 (18.0)	48 (23.5)		31 (15.8)		
Comorbidities						
Congestive heart failure	21 (10.6)	59 (28.9)	0.01	98 (49.7)	0.00	0.02
Hyperlipidemia	29 (14.6)	36 (17.6)		37 (18.8)		
Other minors	43 (21.6)	51 (25.0)		48 (24.4)		
None	106 (53.2)	58 (28.5)		14 (7.1)		
Treatment						
Oral glycemic agent	139 (69.8)	143 (70.1)	0.28	119 (60.4)	0.35	0.55
Insulin	16 (8.0)	14 (6.9)		23 (11.7)		
Combination	44 (22.2)	47 (23.0)		55 (27.9)		
Behavior characteristics						
Self-care activities (days/week)						
1–3	123 (61.8)	91 (44.6)	0.00	15 (7.6)	0.00	0.01
4–7	61 (30.7)	30 (14.7)		29 (14.7)		
None	15 (7.5)	83 (40.7)		153 (77.7)		
Prior diabetes education						
Yes	73 (36.7)	78 (38.2)	0.47	74 (37.6)	0.59	0.64
No	126 (63.3)	126 (61.8)		123 (62.4)		

*BMI* body mass index

^ł^*p* value against control group. * *p* < 0.05. 1 US$ equivalent to 3.88 MYR. N.B: ethnic distribution was excluded from analysis

### Association of demographic with quality of life and health status

Control group have significantly higher mean QOL score (*p *< 0.001) for age, marital status, education status and also economic status as compared to arm 2. However, as compared to arm 1 marital status had similar mean QOL pattern. Findings suggested that several demographic factors reflect glycemic control with low mean QOL score but ex-married/single status was the significant factor for mediating poor glycemic control among participants of arm 2 in comparison between arm 1 and control group. Findings also showed that participants with low economic status had low mean QOL score (*p *< 0.001) regardless to glycemic level in all three groups. Table 2 showed brief distribution pattern of QOL means score and health status with demographic of study population.

**Table 2:** Distribution pattern of quality of life (QOL) and health-status in sociodemographic of study population

Characteristics	Control (n = 199)				Arm 1 (n = 204)					Arm 2 (n = 197)					Arms 1 vs 2
	QOL		Health status		QOL		Health status		*p* ^ł^	QOL		Health status		*p* ^ł^	*p*-value
	Mean	*p*-sig	Mean	*p*-sig	Mean	*p-sig*	Mean	*p*-sig		Mean	*p-sig*	Mean	*p*-sig		
Gender															
Men	13.12	0.35	70.44	0.42	13.02	0.29	71.29	0.25	0.17^#^	13.11	0.65	72.01	0.53	0.22^#^	0.51^#^
Women	12.98		71.11		12.93		70.13		0.23^≠^	12.88		70.18		0.19^≠^	0.66^≠^
Age (years)															
35–44	12.23	0.00	69.43	0.52	11.38	0.00	76.37	0.01	0.00^#^	12.45	0.00	70.11	0.01	0.01^#^	0.03^#^
45–54	10.07		68.89		10.35		67.24		0.00^≠^	10.57		65.29		0.00^≠^	0.04^≠^
55–64	8.73		70.01		8.42		73.13			8.51		63.11			
65–74	9.17		66.89		8.51		66.19			8.72		60.47			
Marital status															
Currently married	12.93	0.00	70.68	0.43	12.65	0.00	69.67	0.89	0.21^#^	11.66	0.00	66.94	0.63	0.00^#^	0.02^#^
Single/ex-married	10.02		68.02		10.23		69.37		0.35^≠^	9.78		68.47		0.00^≠^	0.23^≠^
Education status															
Primary	9.98	0.47	67.96	0.41	9.85	0.00	67.34	0.73	0.00^#^	9.45	0.36	67.23	0.68	0.00^#^	0.41^#^
Secondary	9.93		70.59		11.09		65.23		0.02^≠^	9.31		64.72		0.00^≠^	0.56^≠^
University	9.45		68.11		8.89		64.91			8.13		62.73			
Higher	11.33		75.21		10.21		71.45			9.56		69.62			
Economic status/month															
≤ 1500 US$	8.63	0.00	65.23	0.02	8.93	0.00	66.84	0.31	0.00^#^	8.66	0.21	65.23	0.83	0.00^#^	0.02^#^
> 1500 US$	12.97		74.36		11.29		69.57		0.01^≠^	9.17		66.89		0.00^≠^	0.00^≠^

^ł^*p* value against control group. * *p* < 0.05, ^#^ vs QOL, ^≠^ vs health status. Health status: 0–100 (worst imaginable health to best health)

Mean health state score was significantly higher (*p *< 0.001) among controls with age, education status and economic status as compared to arm 1. Control group showed similar findings with health status as earlier with QOL as compared to arm 2. However, comparison between arms 1 and 2 showed that only age and economics status determine the low mean health state score. Thus finding suggest that moderate to poor glycemic control reflect no influence on mean QOL and health state score with gender, marital status and economic status (Table 2).

### Association of clinical parameters with quality of life and health status

Participants with diabetes history > 10 years exhibits higher mean QOL score among all the three groups. In contrast mean health status score significantly (*p *< 0.001) reduced with the exposure duration of diabetes both within and intergroup assessment (Table 3). Similar pattern was observed with other clinical parameters; BMI, comorbidities and treatment however the significant difference was reported among control vs arms 1 and 2. Intergroup pattern showed no significant difference either on QOL or health status in-between arms 1 and 2 except comorbidities mean health status score slightly (*p *< 0.04) lower in arm 2 as compared to arm 1.

**Table 3:** Distribution pattern of quality of life (QOL) and health-status in clinical parameters of study population

Characteristics	Control (n = 199)				Arm 1 (n = 204)					Arm 2 (n = 197)					ARM 1 vs 2
	QOL		Health status		QOL		Health status		*p* ^ł^	QOL		Health status		*p* ^ł^	*p*-value
	Mean	*p*-sig	Mean	*p*-sig	Mean	*p-sig*	Mean	*p*-sig		Mean	*p-sig*	Mean	*p*-sig		
Duration of diabetes years															
0–5	7.81	0.00	75.70	0.00	8.71	0.02	72.14	0.02	0.01^#^	8.98	0.01	73.45	0.00	0.02^#^	0.86^#^
6–10	9.61		70.51		10.63		70.11		0.78^≠^	10.88		69.45		0.04^≠^	0.74^≠^
> 10	10.88		64.89		10.71		63.80			10.91		61.89			
BMI (kg/m^2^)															
< 18.5/underweight	7.75	0.01	75.25	0.00	8.85	0.74	69.28	0.86	0.03^#^	10.03	0.78	71.04	0.52	0.01^#^	0.44^#^
18.5–24.9/healthy	8.29		69.45		9.12		70.14		0.01^≠^	9.89		70.34		0.01^≠^	0.68^≠^
25.0–29.9/overweight	9.48		65.14		9.58		69.85			10.18		69.25			
Comorbidities															
Congestive heart failure	9.23	0.00	65.18	0.00	9.48	0.00	69.88	0.45	0.76^#^	8.98	0.44	66.34	0.48	0.72^#^	0.61^#^
Hyperlipidemia	7.48		64.89		7.37		70.45		0.01^≠^	6.98		65.62		0.01^≠^	0.04^≠^
Other minors	7.19		70.09		7.88		70.19			7.21		69.45			
None	6.81		75.10		7.01		70.23			7.42		68.61			
Treatment															
Oral glycemic agent	10.49	0.04	70.15	0.00	10.09	0.02	69.39	0.47	0.71^#^	10.40	0.54	69.25	0.21	0.71^#^	0.63^#^
Insulin	9.27		70.84		9.16		67.45		0.01^≠^	8.76		68.34		0.01^≠^	0.25^≠^
Combination	8.84		71.29		8.20		68.24			8.31		67.01			

^ł^*p* value against control group. * *p* < 0.05, ^#^ vs QOL, ^≠^ vs health status. Health status: 0–100 (worst imaginable health to best health)

These demographic and clinical parameters interacted with specific dimensions of life, thus exhibited significant differences on QOL and health status score among different glycemic control groups.

### Association of knowledge and self-care behavior with quality of life and health status

Participants of control group showed significant association with higher mean QOL and health state score (*p *< 0.001) on self-care activities, knowledge of DM and management and prevent daily life activities dimensions. Findings showed that ability to manage positively was significantly associated (*p *< 0.03) with QOL mean score among arm 2 participants only. Findings also suggested that participants with poor glycemic control (arm 2) had significantly higher mean QOL score with knowledge and self-care dimensions as compared to others, however mean health state scores were significantly (*p *< 0.001) lower in all assessment dimensions as compared to controls. Table 4 presented the details of analysis and findings.

**Table 4:** Distribution pattern of quality of life (QOL) and health-status in knowledge and self-care behavior of study population

Characteristics	Control (n = 199)				Arm 1 (n = 204)					Arm 2 (n = 197)					ARM 1 vs 2
	QOL		Health status		QOL		Health status		*p* ^ł^	QOL		Health status		*p* ^ł^	*p*-value
	Mean	*p*-sig	Mean	*p*-sig	Mean	*p*-sign	Mean	*p*-sign		Mean	*p*-sign	Mean	*p*-sign		
Diabetes prevents daily activity															
Never	12.17	0.00	75.27	0.01	10.24	0.00	73.11	0.35	0.00^#^	11.12	0.00	67.91	0.26	0.00^#^	0.23^#^
Sometime	10.09		66.42		12.29		71.79		0.00^≠^	11.60		65.41		0.01^≠^	0.00^≠^
Mostly	9.03		65.37		8.28		71.07			9.25		60.92			
Positive management															
Poor ability	9.22	0.68	64.13	0.01	9.46	0.14	65.51	0.43	0.36^#^	10.46	0.03	63.16	0.87	0.00^#^	0.02^#^
Moderate	9.83		67.01		9.68		64.32		0.02^≠^	9.72		65.39		0.01^≠^	0.73^≠^
Good ability	9.71		72.81		9.57		67.87			10.57		65.98			
Knowledge of DM and its management															
Fair	11.64	0.00	64.17	0.00	11.36	0.00	65.31	0.02	0.00^#^	11.86	0.39	64.13	0.01	0.01^#^	0.56^#^
Above average	9.07		69.36		9.73		66.87		0.00^≠^	11.19		65.20		0.01^≠^	0.43^≠^
Good	9.73		70.39		10.33		71.09			10.38		68.13			
Excellent	12.33		85.32		10.00		73.81			9.35		73.23			
Self-care activities (days/week)															
1–3	10.02	0.00	65.74	0.00	9.89	0.47	66.01	0.00	0.00^#^	11.35	0.02	64.87	0.43	0.00^#^	0.00^#^
4–7	9.23		73.48		9.67		75.01		0.33^≠^	10.11		69.85		0.02^≠^	0.26^≠^
None	10.98		61.93		10.01		60.33			10.89		60.13			
Prior diabetes education															
Yes	10.36	0.02	74.19	0.01	10.53	0.02	69.39	0.03	0.43^#^	11.29	0.36	65.32	0.04	0.00^#^	0.00^#^
No	8.82		66.15		9.32		63.25		0.01^≠^	10.21		61.47		0.01^≠^	0.63^≠^

^ł^*p* value against control group. * *p* < 0.05, N.B: merging Likert-scale for evaluating differences in response. ^#^ vs QOL, ^≠^ vs health status. Health status: 0–100 (worst imaginable health to best health)

### Interdependent relationship with predictors

A generalized linear model method was used to explore the relationship between QOL and health state with the predictors (demographic, clinical, knowledge and behavior dimensions) as provided in Table 5. The combined effect of predictors on QOL and health state using multivariate tests, findings showed duration of diabetes, conditions/comorbidities and diabetes prevents daily life activities were significant predictors of QOL among controls (consistent glycemic control participants), however age, duration of diabetes, BMI, prior diabetes education and ability to manage positively were strong predictors to health state among controls.

**Table 5:** Generalized liner model to estimate effect of dimensions on dependent variable (QOL and health-status) among study population

Characteristics	Control—mean square (F)		Arm 1—mean square (F)		Arm 2—mean square (F)	
	QOL	Health status	QOL	Health status	QOL	Health status
Age	189.72 (28.25)	538.62 (4.98)*	143.72 (21.56)	451.86 (5.49)**	0.98 (0.79)*	386.84 (4.89)**
Economic status/month	0.508 (0.17)	57.964 (0.89)	0.43 (0.08)	55.69 (0.60)	0.12 (0.01)	41.39 (0.44)**
Duration of diabetes	21.83 (4.80)*	495.69 (4.79)**	20.98 (3.30)*	273.72 (3.69)*	18.75 (5.32)*	28.77 (0.29)
BMI	16.84 (3.55)	897.41 (12.13)**	13.53 (2.25)	688.15 (9.65)	0.28 (0.01)	279.88 (4.59)
Comorbidities	8.62 (3.15)*	100.87 (2.14)	5.52 (1.98)*	78.89 (2.46)	0.88 (0.03)*	10.37 (0.18)
Diabetes prevents daily activity	53.34 (15.96)**	109.26 (1.09)	48.91 (14.35)*	102.14 (0.87)	18.71 (1.44)	22.69 (0.49)
Positive management	1.89 (0.42)	498.63 (6.82)***	1.26 (0.36)	256.613 (4.19)	0.15 (0.02)	38.95 (0.41)
Knowledge of DM and its management	2.80 (1.55)	5.89 (0.05)	1.73 (0.95)	2.75 (0.44)	0.65 (0.07)	1.81 (0.02)
Self-care activities	9.68 (6.84)**	351.38 (4.09)	8.59 (4.86)**	291.46 (2.19)*	1.79 (1.27)*	192.14 (2.10)**
Prior diabetes education	48.36 (4.97)*	289.41 (6.12)**	18.701 (2.87)*	459.31 (6.50)*	7.04 (1.56)	667.19 (7.54)**
Corrected model	183.61 (29.13)*	5684.16 (63.78)*	174.40 (38.64)*	4876.08 (60.84)*	152.47 (43.45)*	4067.77 ( (47.54)*
Intercept	2.192 (5.62)**	307.13 (4.55)***	31.78 (4.82)*	284.98 (3.25)**	36.81 (6.33)*	308.35 (4.11)**
R^2^/R^2^_adjusted_	0.487/0.431*	0.351/0.297**	0.358/0.331*	0.309/0.281*	0.396/0.327*	0.173/0.952**

^ł^*p* value against control group. * p < 0.05, ** p < 0.01, *** p < 0.001. ^#^ vs QOL, ^≠^ vs health status. N.B: gender, marital status and treatment were controlled variables. Wilks’s Lambda multivariate testing. Health status: 0–100 (worst imaginable health to best health)

MANOVA models were used to test the overall model significance and also to test the overall individual effects of predictors. Among controls (participants with consistent glycemic control) 48.7% of the variance in total QOL described by clinical and self-care parameters and 35.1% of variance in health state could be explained by clinical, positive management and self-care behavior. Arm 1 participants with moderate glycemic control, 35.8% of variance on total QOL and 30.9% of variance on health state was explained by clinical and self-care behavior. Lastly, arm 2 participants with poor glycemic control, 39.6% of variance on total QOL and 17.3% of variance on health state was explained by demographic and self-care behavior. The *R*^*2*^ values indicated an understanding relationship of predictors with QOL and health state. Hence, demographic and clinical parameters showed strong positive effect on QOL and health state supporting the self-care behavior and diabetes management model (*p *< 0.001). The *F* test of significance showed that demographic and clinical parameters were strong predictors of QOL, whereas self-care activities, comorbidities, ability of positive management and BMI were significant predictors to health state for consistent glycemic control (controls) as compared to poor glycemic control (arm 2) participants (Table 5).

## Discussion

Participants’ age (45 < year), duration of diabetes exposure (10 < years), comorbidities, positive ability to manage disease, low to none barriers to daily life activities and high self-care daily/weekly activities showed positive reflection to QOL and health state among consistent glycemic control group as compared to moderate and poor glycemic control patients’. This showed that increased QOL and improve health state among T2DM with control glycemic levels, as they overwhelmed barriers and challenges in the management of the disease. Previous studies were suggesting similar pattern [20, 21] that self-care behavior significantly improved QOL. But in this study, consistent-to-poor glycemic variability impact on QOL and health state showed that poor glycemic index strongly reduced the QOL and deteriorate health state of the patients’, congruent with the previous studies [22], however this study also found that moderate glycemic index patients showed similar pattern as consistent control participants.

These findings were supported from the past studies; younger age [23], less duration of diabetes [24], diabetes knowledge [15, 25], self-care behavior [10, 14, 18, 26] and comorbidities [27].

Diabetes preventing daily activities were reported by patients age > 55 years, middle age participants without any barrier to daily activities showed positive ability to disease management and high self-care activities. Reduced daily life activities significantly reduced the QOL and health state among T2DM patients [17, 28]. A strong effect was reported with “never” preventing daily living activities to consistent glycemic index with QOL. Both moderate and poor glycemic control participants were significantly reported ‘sometimes’ or ‘moderately’ prevention of daily activities due to diabetes. Increased physical activity contributes to improved glycemic index and subsequently increased QOL [18, 21].

Participants with poor glycemic variability had consistently low QOL for all the dimensions compared to consistent control index patients. This poor QOL prevented participants to manage diabetes positively. Specific domains like age, duration of diabetes, economic status and comorbidities influence relative QOL and health state among arm 2 participants as compared to arm 1 and control. However, the independent predictors might have a contradictory effect on different aspects of QOL and health status. Literature suggested that unexplained emotional problems, concomitant clinical condition and low socioeconomic status cause more restrictions in daily life thus lead decrease QOL and health status among T2DM [29–31].

Good ability to manage diabetes positively contributed to more self-confidence and improve self-care behavior thus less likely to be depresses or anxious. Therefore, good diabetes knowledge, positive attitude and high self-care activities are predictors of adherence to glycemic index and endorse QOL. Consistent glycemic index, high education level, middle age, low duration of diabetes, moderate weekly self-care activities had a significantly higher likelihood of conquering greater QOL scores [14, 16–19, 32].

Family history of diabetes mellitus [28], duration of diabetes [25], comorbidities [29] and medication adherence [13, 14, 30] were also the significant factors for reducing HRQoL among patients with T2DM. Poor HRQOL was reported among oral antidiabetic medications (OAM) patients then insulin users. ED-5D VAS index also reported low values among patients with OAM use only. The understanding of negative impact of T2DM is widely reported among patients [10, 13, 15, 24, 25] but concern raised is treatment modalities may reverse such deteriorating effect or not. A study reported that patients with insulin use in low-middle income countries maintained high QoL in T2DM patients [33], thus recommended early clinical inertia of insulin treatment. Treatment satisfaction and medication adherence are determinants to multiple outcomes; treatment success, HRQoL and care cost [12, 33, 34].

This study reported that controlled glycemic index had higher QOL scores and health state for lower-middle age, low BMI, adherent to self-care activities and least comorbidities. These findings reflected that low duration of diabetes exposure and high education status key factor for self-care behavior. Hence, chronicity of T2DM had a differential impact on QOL and health state in contrast to glycemic variability [10, 20, 21, 25]. Previous studies had reported BMI inverse effect on QOL score among T2DM patients [33, 34].

## Conclusion

This study suggested that poor glycemic index reported low self-care behavior, increase barriers to daily living activities and poor ability to manage diabetes positively. Which leads to poor QOL and decrease health state. Clinical and demographic factors were also influenced QOL and health state with glycemic index variability. Also duration of diabetes exposure and positive self-care behavior are strong predictors of increase QOL and health state in participants with consistent control glycemic index.

### Limitations of the study

1. Lacking medical criteria for secondary clinical parameters determining diabetes mellitus related to comorbid conditions; e.g., renal profiling, liver functioning, hematological findings etc. Age-criterion further limit the proportion of patients’ sample and reduce the study population to 5–10%.
2. Self-care reporting particularly with self-perception may prod the diabetics to answer normatively.
